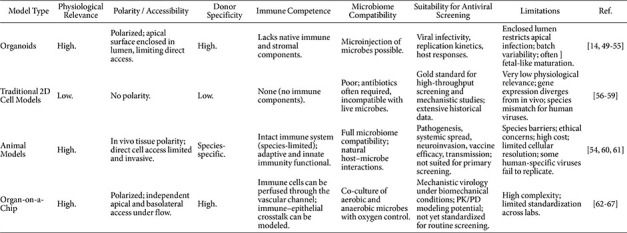# Intestinal Organoids as Models to Study Viruses: Current Application and Future Perspective

**DOI:** 10.4014/jmb.2603.03016

**Published:** 2026-07-07

**Authors:** Wang Ruting, Liang Jiale, Wu Hongxi, Huang Zhenjin, Zhang Ruohan, Song Yuanbo, Zhang Rongxin, Tang Hongzhen, Jiang Feng

**Affiliations:** 1 Graduate School, Guangxi University of Chinese Medicine, Nanning, Guangxi, P. R. China; 2 The Second Affiliated Hospital of Guangxi University of Chinese Medicine, Guangxi, P. R. China

**Keywords:** Intestinal organoids, Organoid culture establishment, Virus model, Virus model comparison, Organoid technology update

## Abstract

Intestinal organoids have emerged as a transformative model system in virology, bridging the gap between conventional cell lines and animal models by recapitulating the complex cellular diversity, three-dimensional architecture, and key functions of the human intestinal epithelium. This review highlights how this technology has enabled groundbreaking studies of enteric viruses, including the successful cultivation of previously uncultivable human norovirus, and has provided critical insights into the infection mechanisms of rotavirus, enterovirus A71, and Severe Acute Respiratory Syndrome Coronavirus 2. We discuss how emerging technologies, such as co-culture systems for host-microbiome interactions, vascularization techniques, and CRISPR/Cas9 gene editing, are being integrated with organoids to create more physiologically relevant microphysiological systems. Despite challenges related to immune component integration and model standardization, intestinal organoids offer a promising platform for elucidating virus-host interactions, advancing antiviral drug screening, and promoting personalized infectious disease research.

## Introduction

Viruses represent the most abundant and genetically diverse group of pathogens, and the gastrointestinal infections they cause continue to impose a major global health burden [1]. Such pathogens are responsible for a wide range of clinical diseases and pose a persistent and serious public health challenge due to their high variability, strong transmissibility, and potential epidemiological risk [2].

Two-dimensional (2D) cultured cell lines have been used for viral propagation since the early 20th century. However, most cell lines are genetically immortalized, cancerous, or otherwise transformed for long-term culture and generally exhibit defects such as dysfunctional innate immune signaling pathways [3]. Since Sato *et al*. successfully generated the first three-dimensional (3D) epithelial organoid from a single leucine-rich repeat-containing G protein-coupled receptor 5 (LGR5) intestinal stem cell in 2009, organoid models derived from human and animal stem cells from a variety of organs have rapidly evolved, greatly advancing basic research, including virology [4]. In this context, intestinal organoids are rapidly becoming an ideal platform for elucidating virus-host interactions due to their unique advantages. Compared with traditional 2D cell lines, intestinal organoids better preserve cellular composition, epithelial polarity, and functional fidelity, making them a highly promising tool for studying enterovirus infection mechanisms, host immune responses, and virus-microbiome-host interactions [5, 6].

This review focuses on key virological findings obtained using intestinal organoids. It also discusses the limitations of intestinal organoids, with particular emphasis on aspects relevant to virological studies, and the emerging strategies being investigated to address these limitations.

## Development of Intestinal Organoids: From Cell Aggregation to Intestinal Organoids

As early as 1907, Henry Van Peters Wilson made the first attempt at *in vitro* regeneration of organisms, demonstrating that dissociated sponge cells could self-organize to regenerate entire organisms [7]. In 1981, researchers first isolated and cultured pluripotent stem cells (PSCs) from mouse embryos, which promoted the rapid development of stem cell research [8]. However, it was not until 1998 that scientists first isolated and cultured embryonic stem cells derived from human blastocysts [9]. Subsequently, the establishment of induced PSCs (iPSCs) through the reprogramming of mouse and human fibroblasts had a profound impact on stem cell research and organoid construction [10, 11]. In 1987, Li *et al*. discovered that mammary epithelial cells grown in EHS extracts could form 3D ducts and luminal structures and, compared with 2D culture, appeared able to synthesize and secrete milk proteins [12]. This study revealed the advantages of 3D culture. Subsequently, Eiraku *et al*. used a 3D aggregation culture method to generate cerebral cortex tissue from embryonic stem cells (ESCs) for the first time, marking a breakthrough in organoid research from 2D to 3D culture models [13]. In 2009, Sato *et al*. published a landmark study showing that single adult intestinal stem cells expressing LGR5 can form 3D intestinal organoids in matrix gel, self-organize, and differentiate into crypt-villus structures without a mesenchymal niche [4]. This organoid model, based on adult stem cells (ASCs), laid the methodological foundation for subsequent research on various organoids.

**Fig. 1:**
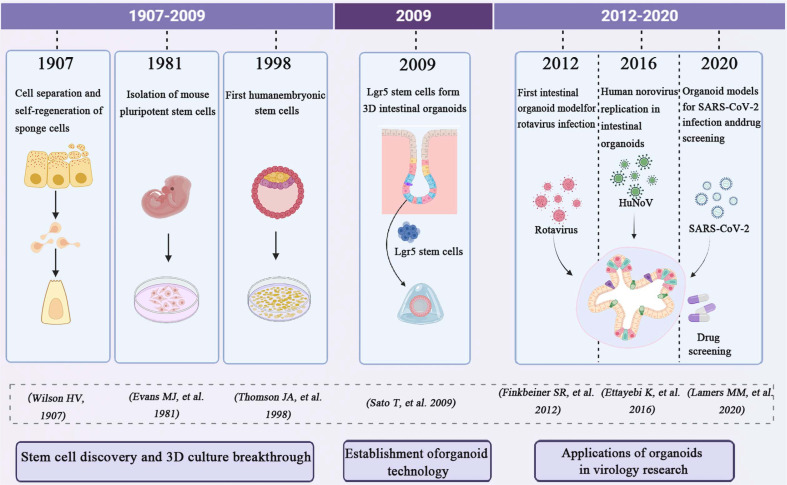
Development of organoids.

With continued technological breakthroughs, organoids have also begun to demonstrate great potential in viral research. In 2012, researchers used organoids derived from embryonic PSCs to study infection by clinical isolates of rotavirus [14]. This marked the first application of intestinal organoid technology to viral research and pioneered the use of organoid models to investigate intestinal viral infections. In 2016, Ettayebi and colleagues pioneered the development of human intestinal organoids (HIOs) capable of supporting norovirus replication. This robust in vitro replication system, applicable to multiple human norovirus (HuNoV) strains, provided insights into strain-specific growth requirements and opened new avenues for norovirus research[15]. At the onset of the Severe Acute Respiratory Syndrome Coronavirus 2 (SARS-CoV-2) pandemic, organoids were used to investigate the virus's culture conditions, infection pathways, and drug screening. In 2020, Lamers *et al*. successfully developed a SARS-CoV-2 intestinal infection model using intestinal organoids, laying a solid foundation for subsequent SARS-CoV-2 research [16, 17]. From norovirus and rotavirus to SARS-CoV-2, the rapid advancement of organoid technology has accelerated and deepened research on human viruses. The developmental history of organoid technology and its major breakthroughs in virology research are illustrated in Fig. 1.

## Characteristics of Intestinal Organoids: A Solid Foundation for Viral Research

Organoids are miniature, simplified models that closely mimic the structure and function of real organs. They form through the self-assembly of stem cells, including PSCs and ASCs, under 3D in vitro culture conditions [18]. Owing to the enhanced self-renewal capacity of stem cells, organoids retain key cell types, spatial architecture, and partial physiological functions of their source tissues [19]. This makes organoids an ideal in vitro model for studying complex intestinal tissues and provides a powerful platform for biological research.

Before discussing their characteristics, it is necessary to clarify several related but distinct intestinal epithelial culture systems used throughout this review. Based on stem cell origin, they fall into two main categories: ASC-derived organoids (also called enteroids) and PSC-derived organoids, including those derived from embryonic stem cells or induced PSCs [20, 21]. HIOs generally refer to organoids derived from PSCs, whereas human intestinal enteroids (HIEs) specifically denote ASC-derived organoids from intestinal crypts [22, 23]. Human small intestinal organoids (hSIOs) are established from primary gut epithelial stem cells, can be expanded indefinitely in 3D culture, and contain all proliferative and differentiated cell types of the in vivo epithelium [24]. Additionally, organoid-derived monolayers are 2D cultures obtained by dissociating 3D organoids and growing the cells as a polarized monolayer on permeable supports, which facilitates apical and basolateral infection studies and high-throughput screening [25, 26]. Distinguishing these models is critical for virology research, because HIEs better retain region-specific epithelial properties and support replication of some viruses (*e.g.*, HuNoV) that do not grow well in PSC-derived HIOs. Conversely, PSC-derived HIOs offer advantages for studying developmental stages and genetic manipulation [27, 28]. Recognizing these differences allows researchers to select the most appropriate system for a given virus and research question.

### Cell Type Diversity

**Fig. 2:**
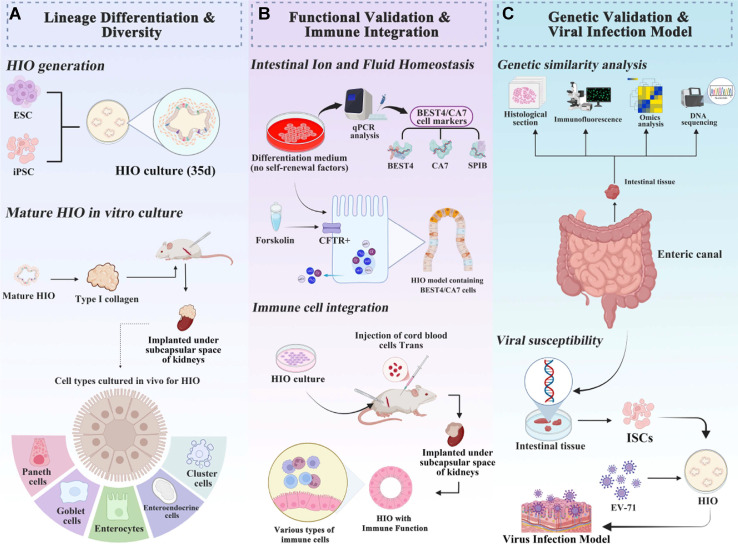
Characteristics of intestinal organoid construction. (**A**) ESC/iPSC-derived HIOs matured in the subcapsular space of mouse kidneys contain all major intestinal epithelial cell types, including enterocytes, goblet cells, Paneth cells, enteroendocrine cells, and cluster cells. (**B**) In differentiation medium lacking self-renewal factors, HIOs can generate rare BEST4/CA7^+^ cells that highly express CFTR; the CFTR agonist forskolin interferes with ion transport, thereby confirming that ion and fluid transport depend on CFTR. HIOs transplanted into humanized NSGS mice recruit various human immune cells, mimicking intestinal immune function. (**C**) Patient-derived HIOs preserve the donor's native genetic background and can be infected with enteric viruses (EV-A71) for personalized pathogenesis studies. CFTR: cystic fibrosis transmembrane conductance regulator. HIOs: human intestinal organoids. ESCs: embryonic stem cells. ISCs: intestinal stem cells. iPSCs: induced pluripotent stem cells.

In earlier studies, although intestinal organoids showed substantial structural and cellular similarities to the fetal intestine, they lacked the molecular and structural characteristics of the adult intestine. For example, villous structures were too short, crypts were absent, and there was no evidence of functional intestinal stem cells (ISCs) or associated immune functions [29]. To overcome these shortcomings, researchers have implemented a series of improvements. For example, Watson *et al*. embedded intestinal organoids in type I collagen and transplanted them into the subcapsular space of mouse kidneys, allowing them to mature and grow for 6 weeks before retrieval [30]. The implanted tissue resembled natural human intestine, featuring crypt-villus architecture and an underlying layered submucosa comprising the lamina propria, muscularis mucosae, and submucosa, along with an external smooth muscle layer [30]. Compared with in vitro culture, in vivo transplanted tissues exhibited greater maturity and differentiation; all major intestinal cell lineages, including enterocytes, goblet cells, Paneth cells, enteroendocrine cells, and cluster cells, were located in the appropriate regions of the crypt-villus axis, and Paneth cells were situated as expected at the crypt base rather than dispersed throughout the epithelium [30]. In a recent study, researchers achieved greater cellular diversity within organoids by enhancing stemness, thereby unlocking multilineage differentiation capacity and potential [31]. This approach avoided reliance on lineage-restricted progenitor cells or direct induction of differentiation [31]. The method resulted in accelerated growth and increased cellular diversity, demonstrating advantages over existing protocols (Fig. 2A).

With continuous innovations in cultivation techniques, intestinal organoids increasingly resemble natural intestinal tissue in both structure and function. Intestinal organoids are, therefore, expected to have broad applications in areas such as intestinal development, disease modeling, and drug research and development.

### Similarity of Key Functions

Through technologies such as co-culture and gene editing, organoid models have made significant advances in simulating key physiological processes, including intestinal ion channel function and the homeostatic balance between the microbiota and host, thereby providing powerful tools for precision medicine research (Fig. 2B).

Within the intestine, various cell types work together to maintain electrolyte and water balance [32]. Cystic fibrosis transmembrane conductance regulator (CFTR) is a key Cl−/HCO3− channel, and many pathogens cause diarrhea by targeting this channel to disrupt ion transport [33]. BEST4/CA7 cells are the only CFTR-high-expressing cells in the human small intestine [34]. To investigate their role in the pathogenesis of diarrhea, Wang *et al*. successfully established a HIO model containing BEST4/CA7^+^ cells [34]. Using a specific differentiation medium (i.e., surrogate WNT, +14 Noggin, EGF, A83-01, SB202190, prostaglandin E2, and nicotinamide), removal of self-renewal factors promoted robust differentiation of stem cells into multiple cell lineages, including rare cell types [34]. Following administration of CFTR activators such as forskolin, organoids swelled due to luminal fluid accumulation, mimicking the pathological fluid secretion observed in disease states [34]. These findings confirm that, through a series of technical approaches, organoids can closely mimic intestinal ion and fluid homeostasis.

Similarly, following subcapsular transplantation of intestinal organoids into the kidney, Bouffi *et al*. characterized human immune cells infiltrating the lamina propria and epithelium of the intestinal organoids [35]. The results indicate that developing intestinal organoids and cells inoculated into the lamina propria attract and activate immune precursors by expressing specific signals. They influence and shape the formation of immune aggregates, serving as the foundation for subsequent lymphoid tissue development. This process establishes intestinal organoids as in vivo models of functional human immune tissues [35]. Notably, the size of intestinal organoids does not correlate with the percentage of human immune cells in the peripheral blood of humanized mice [35].

### Genetic Similarity

The core advantage of intestinal organoid models lies in their exceptional genetic and pathological fidelity, which positions them as a vital bridge between basic research and clinical translation [36]. Organoids can be derived directly from a patient's ASCs or iPSCs, fully preserving the donor's individual genetic background, including critical information such as single-nucleotide polymorphisms and epigenetic modifications, thereby enabling precise recreation of specific pathological phenotypes in complex diseases such as inflammatory bowel disease, cystic fibrosis, and colorectal cancer [37-40]. This characteristic is particularly important in virology research, especially when studying pathogens with strict species specificity. Taking enterovirus 71 (EV71) of the family Picornaviridae as an example, humans are their only natural host, posing significant challenges to the establishment of an ideal animal model. Existing models, such as mice or non-human primates, fail to comprehensively capture human disease characteristics: some lack face validity because symptoms differ from those in humans, whereas others lack construct validity because pathogenic mechanisms differ from those in humans)[41]. Notably, studies in non-human primates reveal significant differences among monkey species in tissue tropism, primary viral replication sites, and clinical manifestations following EV71 infection. This further underscores the critical role of genetic background in disease expression and highlights the urgent need to develop personalized medical models [41]. Patient-specific stem cell-derived intestinal organoids serve as an ideal platform to address this challenge. Their highly faithful genetic characteristics provide an irreplaceable experimental system for in-depth investigation of host-specific viral infection mechanisms (Fig. 2C).

## Advantages of Organoids Over Traditional Models in Viral Research

Given the complexity of the intestinal microenvironment and the difficulty of obtaining human tissue, determining the processes that maintain intestinal homeostasis has always been challenging. Traditional virus research models, such as primary cell cultures and passaged cell lines, offer advantages of easy access, straightforward operation, and relatively low cost, making them suitable for rapid virus isolation and amplification [42]. However, these models have significant limitations. For example, they are typically 2D monolayer cultures that cannot replicate the complex 3D structure and cellular diversity of human tissues; most are derived from tumors, and their genetic background and physiological functions differ significantly from those of normal tissues [43]. Although animal models provide a complete in vivo environment, they differ from humans at the species level [44, 45]. Furthermore, stress conditions imposed during experimental procedures can easily introduce errors into tissue samples, making it difficult to objectively and accurately obtain corresponding data [44]. This often leads to suboptimal translation of research findings into clinical practice. These limitations hinder progress in areas such as in-depth studies of viral pathogenic mechanisms and host responses in physiologically relevant environments. Therefore, building on existing research, it is necessary to develop more advanced biological model systems and strike a balance between experimental practicality and biological fidelity to more accurately assess the potential for cross-species transmission of viral pathogens between humans and animals [46].

The emergence of intestinal organoid technology has had a significant impact on this field [47]. Using ASCs, researchers have cultivated 3D micro-organs in vitro that contain all key intestinal epithelial cell types and closely mimic the physiological structure and function of the human intestine [48].This technology effectively addresses the shortcomings of traditional cell models in terms of biological fidelity and the genetic similarity of animal models, providing a superior platform for studying viral cell tropism, replication kinetics, and host-pathogen interactions (Table 2) [44].

## Intestinal Organoids: A Highly Promising Model for Virus Research

Intestinal organoid models closely recapitulate the 3D structure and key physiological functions of the human intestine, thereby overcoming the limitations of traditional cell lines in related research [68]. Using this platform, researchers can establish more precise models of enteroviruses and more accurately elucidate viral invasion mechanisms, host-pathogen interactions, and immune response processes. This has substantially advanced both fundamental research on enteric viruses and the development of antiviral strategies.

### Human Norovirus

HuNoV is highly contagious and replicates to extremely high levels in the human intestine. Since the discovery of HuNoV in the 1970s, researchers have used numerous cell lines to attempt to culture HuNoV-infected cells, but the results have been unsatisfactory [69, 70]. This technical challenge raises a fundamental biological question: What specific characteristics of the intestinal environment are required for HuNoV replication, and can these characteristics be replicated in laboratory models?[Table T1]

In 2016, Ettayebi *et al*. developed the first stable culture system supporting in vitro replication of multiple HuNoV genotypes based on human intestinal stem cell-derived enteroids (HIEs). Using a 2D HIE monolayer infection protocol, multiple HuNoV strains, including GI.1, GII.3, GII.4, and GII.17, replicated in HIEs under bile acid-dependent conditions [15]. This intestinal organoid model, generated using in-house proliferation (BCMp) and differentiation (BCMd) media, demonstrated enhanced HuNoV culture capacity in fecal samples containing a broader spectrum of HuNoV strains [54]. In another study, researchers used CRISPR/Cas9 technology to introduce the FUT2 gene into an HIE cell line that did not originally support viral replication, successfully establishing a new organoid subline, J4FUT2-KI HIE, that was susceptible to norovirus. This confirmed that FUT2 expression is a key determinant of HuNoV binding and replication, further highlighting the importance of the host genetic background [71]. Subsequently, more robust and standardized human norovirus organoid models were developed, further improving the reproducibility and efficiency of viral replication across laboratories and genotypes [54]. Overall, these findings address key biological questions: HuNoV fails to replicate in conventional cell lines because these cells lack a differentiated epithelial phenotype, an appropriate bile acid profile, and key host factors, such as FUT2, that are naturally present in stem cell-derived intestinal organoids.

After successfully establishing a model of norovirus infection, the researchers were able to obtain previously inaccessible insights. In a study investigating the susceptibility of HuNoV strains GII.4 Sydney [P31] and GII.3 [P21] in different intestinal segments, researchers cultured HIEs using intestinal tissues from the duodenum, jejunum, ileum, and colon obtained from four independent organ donors [72]. The results demonstrated that GII.4 and GII.3 replicated in small-intestinal HIE lines, whereas colonic HIE lines did not support replication of either strain, indicating segment-specific differences in susceptibility to HuNoV infection among HIE lines [72]. Notably, all intestinal segments from one independent donor were completely nonpermissive for GII.3 replication, whereas GII.4 replicated normally in the small-intestinal segments from the same donor [72].Viral replication efficiency varied across intestinal segments and donors, indicating differences in receptor expression, innate immune responses, or other host factors. Ayyar *et al*. used HIOs to show that the GII.4 genotype enters cells through a unique pathway involving CLIC (chloronectin-dependent internalization), acid sphingomyelinase-mediated lysosomal exocytosis, and membrane wound healing, a mechanism distinct from classical endocytosis that may help explain the virus’s resistance to certain antiviral strategies [73].

Despite these transformative advances, important limitations remain. Even the most optimized HIE systems still lack key components of the native gut microenvironment, including resident immune cells, gut microbiota, and neural inputs. Consequently, questions regarding how these elements modulate HuNoV pathogenesis or contribute to viral evasion of host immunity cannot yet be fully addressed. Furthermore, although the system has been used for drug testing—for example, nitazoxanide (NTZ) was evaluated in transgenic J4FUT2-KI HIE lines and showed no significant suppression across four distinct HuNoV genotypes—no antiviral has demonstrated consistent efficacy [74]. The cost, technical variability, and relatively low throughput of organoid cultures also make large-scale screening impractical at present. Thus, although HIEs have revolutionized HuNoV research by identifying the fundamental requirements for viral replication, the field still lacks standardized, high-throughput platforms that integrate immune and microbial components to address more complex questions related to host defense and therapeutic intervention.

### Rotavirus

Rotavirus is a major cause of childhood diarrhea worldwide. Owing to the lack of precise viral models, studies of infection mechanisms and drug development remain constrained [75, 76]. A longstanding technical challenge is that clinical rotavirus isolates from stool samples grow poorly, if at all, in conventional immortalized cell lines; they often require multiple rounds of blind passage in primary cells before adapting to these artificial environments, and even then the success rate is low [77]. This raises an important question: Is there a more physiologically relevant intestinal model that can support direct infection by clinical rotavirus isolates without prior adaptation?

The advantages of intestinal organoids over traditional cell lines became evident in the work of Finkbeiner and colleagues, who used HIOs to study both laboratory strains and unpassaged clinical isolates [14]. When HIOs were inoculated with rotavirus-positive stool samples comprising multiple genotypes, including G9P [8], G1P [8], G3P [8], G2P [4], and G3P [untypeable], most isolates replicated efficiently. At 24 h postinfection, viral VP7 RNA levels increased more than 10-fold in most samples; by 48 h postinfection, all samples showed at least a 10-fold increase, and several exceeded 100-fold[14]. Notably, this was achieved without prior adaptation of the clinical isolates to cell culture. Thus, the organoid model clarified why conventional cell lines fail: they lack the differentiated epithelial phenotype and appropriate host factors preserved in HIOs. The ability to directly test clinical isolates without laborious blind passage represents a clear advantage over traditional systems.

Beyond supporting viral growth, organoids have allowed researchers to reexamine how the local intestinal environment influences rotavirus infection, a question that is difficult to address with conventional cell cultures because they lack tissue-level oxygen gradients and 3D architecture. Previous studies suggested that hypoxic conditions might enhance viral production through mechanisms such as altered cellular metabolism [78-80]. Jacobs and colleagues directly tested this hypothesis using rotavirus-infected intestinal organoids cultured under normoxic (20% O_2_) versus hypoxic (1% O_2_) conditions[81]. They found that hypoxia promotes rotavirus replication by upregulating protein phosphatase PP2A and its subunit PPP25RB, which in turn reduces phosphorylation of TBK1 and IRF3, leading to suppressed interferon production [81-83]. This study provided a mechanistic explanation for why hypoxic intestinal tissues may be more permissive to rotavirus, a finding that would have been difficult to obtain in conventional 2D cultures, where oxygen levels are uniform and cell polarity is disrupted. The organoid model has also been used to validate antiviral candidates.

Zhang *et al*. observed that metformin hydrochloride inhibits rotavirus replication in 2D cell lines and further confirmed this effect in rotavirus-infected intestinal organoids, in which viral mRNA levels and replication were significantly reduced[84]. Although this cross-validation strengthens the credibility of the finding, it also highlights a current limitation: the organoid system remains too costly and variable for primary high-throughput screening, so initial identification of drug candidates still relies on conventional cell lines [85].

### Enterovirus A71 (EV-A71)

EV-A71 is a major cause of hand, foot, and mouth disease, particularly among children in the Asia-Pacific region[86]. Many cancer cell lines, such as rhabdomyosarcoma and colonic adenocarcinoma (Caco-2) cells, have been used to test candidate anti-EV-A71 drugs [87]. However, these nonphysiological models do not fully reflect the differentiation status, polarity, and host-restrictive factors of human intestinal epithelial cells. This raises the question: Can more representative intestinal models, such as stem cell-derived organoids, be used to more accurately elucidate the mechanisms of EV-A71 intestinal infection and the determinants of its neurotropism?

Tsang *et al*. addressed this question by constructing HIOs from small-intestinal tissue obtained during gastrointestinal surgery [87]. They characterized the replication kinetics of several enteroviruses, including EV-A71, CVB2, PV-3, and EV-D68, within HIOs [87]. Unlike immortalized cell lines, HIOs retain the cellular complexity and regional identity of the human intestine. The experiments showed that HIOs permit productive replication of EV-A71, CVB2, and PV-3, whereas EV-D68 replicated poorly, consistent with clinical observations that EV-D68 primarily targets the respiratory tract rather than the gut [87, 88]. This concordance between in vitro organoid data and clinical tropism illustrates a key advantage: organoids can help distinguish viruses with different natural routes of infection, something homogeneous cell lines often fail to do because they lack tissue-specific cues.

With regard to neurotropism, a longstanding question is which viral determinants drive EV-A71invasion of the central nervous system. Previous studies have shown that mutations in the receptor-binding capsid protein VP1 are potential drivers of EV-A71 neurotropism; the positive charge generated by glutamine (Q) at VP1-145, together with other sites, including lysine residues at positions 97, 98, 162, 242, and 244, constitutes the primary heparan sulfate proteoglycan (HSPG)-binding site [89, 90]. Using monolayer cells derived from human fetal organoids, Aknouch *et al*. compared two clinical isolates, C1-480-Q (VP1-145 glutamine) and C1-1185-E (VP1-145 glutamic acid) [91]. They found that the Q-containing isolate replicated more efficiently and, importantly, could infect cells from both the apical and basolateral sides, whereas the E-containing isolate infected only the basolateral side. Pretreatment with low-molecular-weight heparin, which blocks HSPG binding, markedly reduced infectivity of both isolates [91]. These findings suggest that HSPG-mediated attachment via VP1-145Q facilitates efficient entry from the apical surface, potentially allowing the virus to breach the intestinal barrier and spread systemically [91]. Thus, the organoid-derived monolayer model clarified how a single amino acid change alters viral tropism and route of entry, information that would have been difficult to obtain using conventional cell lines because they often lack proper apical-basal polarity and relevant receptor expression.

Despite significant progress, several limitations remain. First, the study by Aknouch and colleagues used 2D monolayers derived from fetal organoids rather than fully 3D organoids. Although monolayers provide convenient access to both cell surfaces, they lack the crypt-villus architecture and complex luminal environment that may influence infection *in vivo* [92]. Second, current models do not include a functional blood-brain barrier or neural components, limiting direct investigation of how the virus travels from the gut to the central nervous system. Finally, as with other organoid systems, variability among donor-derived lines and relatively high culture costs hinder large-scale antiviral screening[85]. Therefore, although intestinal organoid-based models have provided valuable insights into EV-A71 intestinal infection mechanisms and the role of VP1-145Q, future efforts should focus on integrating neural elements and developing more standardized, higher-throughput platforms to better understand neuroinvasion and accelerate drug development.

### SARS-CoV-2

The emergence of severe SARS-CoV-2 triggered an unprecedented public health crisis. SARS-CoV-2 infection is primarily associated with respiratory dysfunction, but the gastrointestinal tract is also an established target organ in coronavirus disease 2019. Up to 60% of patients experience gastrointestinal symptoms, such as diarrhea, vomiting, abdominal pain, and loss of appetite [93-96]. These findings underscore the need to establish suitable intestinal infection models to determine whether SARS-CoV-2 can infect and replicate in human intestinal epithelial cells.

To elucidate the pathogenesis of SARS-CoV-2 and support subsequent drug development, Lamers *et al*. investigated SARS-CoV-2 infection in intestinal organoid models [16]. They exposed hSIOs grown under four distinct culture conditions—expansion medium (EXP), differentiation medium (DIF), addition of BMP2/4 to DIF (DIF-BMP), and enteroendocrine cell conditions (EECs)—to SARS-CoV-2. Quantitative RT-PCR and live virus titration in VeroE6 cells demonstrated that SARS-CoV-2 effectively infected all four hSIO types [16]. Compared with differentiated organoids (DIF and DIF-BMP), organoids cultured in EXP demonstrated significantly greater viral replication, suggesting that enterocyte progenitors are the primary viral targets, as hSIOs grown in EXP primarily consist of stem cells and intestinal progenitor cells [16]. Subsequent studies also attempted to culture SARS-CoV-2 in intestinal organoids derived from Jamaican fruit bats, but only limited viral replication was observed, which may reflect species specificity [97].

Beyond establishing basic infectivity, organoids have helped explain why susceptibility varies among individuals and across intestinal regions. Previous studies have shown that intestinal epithelial cells strongly express the SARS-CoV-2 receptor angiotensin-converting enzyme 2 (ACE2) and the transmembrane proteases TMPRSS2 and TMPRSS4, which facilitate viral entry into host cells [98]. Using organoid-derived monolayers from different intestinal segments of multiple donors, with and without inflammatory bowel disease, Jang *et al*. found substantial variability in susceptibility, and ACE2 expression levels correlated most strongly with infectivity. Importantly, donor-specific differences in ACE2 expression were preserved in organoids, mirroring those observed in the original tissue sections [99]. This demonstrates that organoids can retain individual genetic and epigenetic signatures, providing a platform to study host-dependent variation, an advantage over conventional cell lines derived from single donors or pooled sources [99].

The SARS-CoV-2 Omicron variant, which emerged in late 2021, rapidly replaced the Delta variant to become the dominant strain worldwide. Omicron also causes gastrointestinal symptoms in some patients[100]. These findings prompted further investigation of the relationship between the SARS-CoV-2 Omicron variant and the gastrointestinal tract. Previous studies have indicated that Omicron has altered its cell-entry mechanism, relying less on type II transmembrane serine proteases (TTSPs) and more on endosomal cathepsins, such as cathepsins B and L [101, 102]. To test this hypothesis, Mykytyn *et al*. used CRISPR/Cas9-based HIO models to compare viral replication after knockout of TMPRSS2, an important TTSP family member, versus knockout of cathepsin L or cathepsin B [103]. They found that deletion of cathepsin L or cathepsin B had no negative effect on replication [103]. These findings indicate that TTSP inhibition remains a viable antiviral strategy against current variants, and that the organoid model provides a physiologically relevant setting for testing entry mechanisms without relying on overexpressed receptor systems in transformed cell lines.

However, most current SARS-CoV-2 studies focus on acute infection, and evidence remains insufficient regarding the ability of organoids to simulate persistent or recurrent infection, the latter of which has been observed in some patients. Future research will, therefore, need to integrate immune and vascular components to investigate systemic pathogenesis and to develop more reliable models for antiviral testing.

Emerging Technologies Empower Next-Generation Gut Organoid Virus Models

Although traditional intestinal organoid models have demonstrated significant potential in virology research, they still have certain limitations, such as the absence of immune components, difficulty simulating physiological fluid dynamics, and the complexity of host-microbiota interactions. These limitations have prompted researchers to combine organoids with other cutting-edge bioengineering technologies, giving rise to next-generation models with enhanced functionality. These emerging technologies aim to transform static, relatively simplified organoids into dynamic, near-complete microphysiological systems, thereby more authentically recreating the human intestinal microenvironment in vitro.

### Human Intestinal Microbiota Culture

**Fig. 3:**
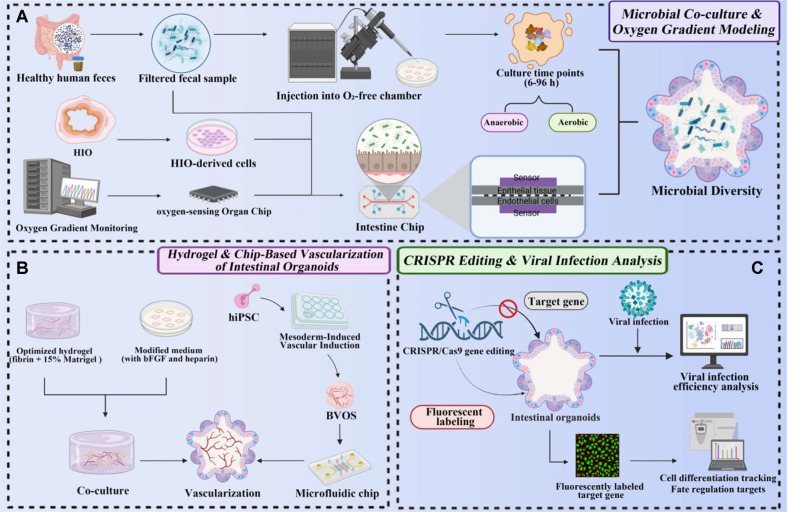
(**A**) Human fecal microorganisms are microinjected into HIOs or seeded into an oxygen-gradient Intestine Chip, enabling stable colonization and real-time monitoring of host-microbiota interactions. Microbiota extracted from human feces are introduced into the chip to generate an Intestine Chip that simulates the human intestinal microbiome. (**B**) An optimized hydrogel (fibrin + 15% Matrigel) and modified medium (bFGF, heparin) support vascular network formation around HIOs. Alternatively, hiPSC-derived blood vessel organoids (BVOs) are integrated into microfluidic chips to generate vascularized organoids-on-a-chip. (**C**) Knockout of target genes in intestinal organoids identifies essential host factors for viral infection. Fluorescent labeling further allows tracking of cell differentiation and fate regulation. bFGF: basic fibroblast growth factor. hiPSCs: human induced pluripotent stem cells. BVOs: blood vessel organoids.

Microbiota, composed of host-specific symbiotic microorganisms, plays a central role in human health and disease [104]. Although the human microbiome colonizes the mucosal surfaces of various tissues, the gastrointestinal tract supports the greatest number and diversity of microorganisms [105]. Aerobic and anaerobic gut symbiotic microorganisms are crucial for maintaining normal nutrient absorption, drug metabolism, and immune homeostasis in the host and form a key biological barrier against pathogen invasion [106]. The gut microbiome and viral communities exhibit close interactions [107]. Symbiotic microorganisms not only indirectly influence viral susceptibility through mechanisms such as competition for colonization sites and secretion of antimicrobial substances, but also constitute a major component of the gut viral community [108]. In previous studies, analyses of crosstalk between the microbiome and intestinal epithelium relied almost exclusively on genomic or metagenomic analyses of in vivo samples. This limitation stems primarily from the absence of experimental models capable of reconstructing complex symbiotic microbial communities *in vitro* [109]. This has prompted researchers to focus on in vitro co-culture techniques for intestinal epithelium and microbiota (Fig. 3A).

The high-throughput organoid microinjection system enables efficient and reproducible delivery of cargo into the lumen of intestinal organoids [53]. It combines a microinjection device with the CVis algorithm and microculture arrays for high-content sampling of inert and biological cargoes injected into organoid lumens [53]. Microinjection of fecal samples into the colonic lumen demonstrated effective fecal microbiota transplantation, with the microbial community persisting for 4 days [53]. The experimental Intestine Chip system is an intestinal epithelial-microbial co-culture platform that supports dynamic interactions between live, mucus-producing human intestinal epithelial cells and their directly corresponding complex human aerobic and anaerobic symbiotic gut microbial communities [110]. The dual-channel organ-on-a-chip system in this study is a microfluidic device incorporating HIO technology. Fabricated from optically transparent, flexible polydimethylsiloxane polymer, it enables oxygen concentration monitoring and incorporates a dedicated anaerobic chamber [110]. This design establishes a physiologically relevant intestinal oxygen gradient within the gut chip. The system enables co-culture of human intestinal epithelium with specialized anaerobic bacteria (*Bacillus fragilis* strain NCTC 9343) on a chip [110]. After inoculation of the human microbiome cultivated from 13 distinct media types onto the intestinal chip, 11 well-defined genera were successfully detected, maintaining high microbial diversity and surviving up to 5 days [110].

### Optimization of Vascularization Techniques for Intestinal Organoid Models

The vascular system is crucial for maintaining organ homeostasis and metabolism. Vascularization of intestinal organoids enhances their growth and maturation[111]. However, traditional intestinal organoid models lack functional vascular networks, limiting their ability to simulate systemic physiological and pathological processes [112, 113]. For example, they cannot reproduce the critical processes by which viruses breach the epithelial barrier in living organisms, enter the bloodstream, spread to distant organs, and trigger migration of immune cells from blood vessels to the site of infection. The development of intestinal organoid models with vascular structures not only fills a gap in in vitro models for simulating systemic infections but also provides a more reliable platform for screening and evaluating antiviral drugs and vaccines (Fig. 3B).

To improve vascularization in intestinal organoids, Wen *et al*. refined the modeling approach [114]. A finely tuned hydrogel was developed by incorporating plasmin inhibitors and 15% Matrigel into fibrin [114, 115]. The culture medium for co-cultured endothelial cells and organoids was modified with basic fibroblast growth factor and heparin [114]. By combining the finely tuned hydrogel with the modified medium, vascular networks and organ-like vessels were successfully formed without fibroblasts [114]. Subsequently, integration of thick collagen fiber bundles into the system guided vascular network formation and enhanced interactions between the vascular network and the organ [114]. Quintard *et al*. developed a chip platform that enables vascularized culture of multiple biological tissues through an innovative microfluidic device and a convenient chip-loading process [113]. The reliability of this system was validated using both spheroid models constructed from human fibroblasts and endothelial cells and 3D blood vessel organoids (BVOs) derived from human-induced PSCs (hiPSCs) [113, 116].These advances significantly overcome common obstacles in organoid growth, maturation, and organ-on-a-chip technology.

### Application of CRISPR/Cas9 Gene Editing Technology in Organoids

The CRISPR/Cas9 system is a gene-editing tool derived from the adaptive immune system of bacteria that enables precise modification of the host genome [117]. In recent years, integration of this technology with organoid models has advanced virology research by enabling precise genetic manipulation within organoids that closely mimic the physiological environment of the human body [118, 119]. Through genome-wide CRISPR screening strategies, researchers have systematically identified key human genes involved in viral replication, thereby pinpointing potential therapeutic targets against related viruses (Fig. 3C).

In a study on SARS-CoV-2, researchers used CRISPR/Cas9 gene editing to deplete TMPRSS2 or TMPRSS4 expression in human duodenal intestinal-like cells to determine the functional roles of TMPRSS serine proteases in promoting SARS-CoV-2-mediated infection of primary human intestinal epithelial cells [120]. The results confirmed that TMPRSS2 and TMPRSS4 promote SARS-CoV-2 infection in intestinal models[120]. Overall, these studies highlight the physiological relevance of organoid models for characterizing virus-host interactions and demonstrate the potential of CRISPR/Cas9 technology for antiviral therapeutics [121]. Notably, CRISPR/Cas9 technology can also help reveal the roles of different intestinal tissue cells in physiological processes within highly human-like intestinal organoids, as well as the key roles of different pathways in regulating cell fate [118, 122-124]. Nevertheless, application of CRISPR/Cas9 in organoids still requires careful optimization to minimize off-target effects and cellular stress.

## Conclusion

Intestinal organoid models have become a valuable platform for viral research due to their unique ability to closely mimic the complex structure and function of the human intestine. Their high fidelity in reproducing cellular diversity, spatial structural integrity, and key physiological functions enables successful replication of pathogens previously difficult to cultivate in vitro, such as HuNoV. This breakthrough offers important opportunities for fundamental virology research, studies of host-immune interactions, and antiviral drug screening. Emerging technologies continue to enhance organoid models, advancing them from static cultures toward dynamic integrated systems. For instance, microphysiological systems replicate the dynamic physiological environment of the intestine by introducing fluid shear forces and mechanical stress; co-culture techniques involving immune cells and specific microbial communities create more complex systems for studying host-microbe-virus interactions; and advances in vascularization technology pave the way for simulating viral hematogenous transmission and systemic immune responses.

However, this technological system still faces numerous challenges. Existing models still have shortcomings in immune component integrity, microbial community stability, and functional maturity; heterogeneity among organoids from different batches poses challenges for experimental standardization; and most models remain unable to fully replicate all characteristics of the adult human gut. These limitations have, to some extent, constrained data reproducibility and translational potential. Looking ahead, advancement of intestinal organoid models will require deep integration and collaborative innovation across disciplines. The primary task is to establish standardized, automated organoid culture and analysis workflows to overcome batch-to-batch heterogeneity, thereby laying the foundation for high-throughput drug screening and large-scale biomedical applications. Significant breakthroughs are also needed to overcome technical bottlenecks in constructing complex microenvironments. By systematically integrating vascular networks, neural innervation, and immune components into organoids, researchers can simulate a more physiologically relevant in vitro human-on-a-chip system, thereby enhancing the model's physiological relevance and predictive value. At the same time, use of patient-specific induced PSCs to construct personalized intestinal organoid models holds promise for disease modeling and evaluation of individualized treatment strategies, thereby advancing precision medicine. In summary, with continued technological innovation and effective cross-disciplinary collaboration, intestinal organoid models may further fulfill their potential in elucidating viral pathogenic mechanisms, developing antiviral drugs, and studying control strategies for emerging infectious disease control. This progress could help bridge the gap between basic research and clinical translation.

## Figures and Tables

**Table 1 T1:** Comparison of various models.